# Pathogenic characteristics and factors associated with multidrug-resistant bacterial infections after perianal abscess surgery

**DOI:** 10.3389/fsurg.2026.1856200

**Published:** 2026-08-04

**Authors:** Bingjie Wang, Xujing Wei, Wanli Ma, Xiangsheng Wang, Binghui Hu, Yefang Zhao

**Affiliations:** 1 Department of Proctology, Xingtai People’s Hospital, Affiliated Hospital of Hebei Medical University, Xingtai, Hebei, China; 2 Department of Obstetrics and Gynecology, First Hospital of Hebei Medical University, Shijiazhuang, Hebei, China; 3 Department of Obstetrics and Gynecology, Xingtai People’s Hospital, Affiliated Hospital of Hebei Medical University, Xingtai, Hebei, China

**Keywords:** associated factor, multi-drug resistant bacteria, perianal abscess, retrospective study, wound culture

## Abstract

**Objective:**

This study aims to describe the pathogenic characteristics of multidrug-resistant (MDR) bacterial infections after perianal abscess surgery and explore its associated factors.

**Methods:**

This retrospective study included 246 adult patients who underwent perianal abscess surgery at our hospital between June 2022 and June 2025. All patients underwent postoperative wound secretion bacterial culture and antimicrobial susceptibility testing. The primary endpoint of this study was the detection of MDR strains in culture, rather than the diagnosis of clinical infection based solely on culture results. Based on culture results, patients were categorized into an MDR culture-positive group (*n* = 78) and a non-MDR culture-positive group (*n* = 168). Baseline characteristics, disease-related features, treatment-related factors, and clinical outcomes were compared between the two groups. Logistic regression analysis was performed to identify factors associated with MDR culture positivity.

**Results:**

Among the 246 patients, 78 (31.71%) were positive for MDR strains. A total of 96 pathogenic isolates were identified, including 62 Gram-negative bacteria (64.58%). The predominant pathogens were *Escherichia coli*, *Staphylococcus aureus*, and *Pseudomonas aeruginosa*. Multivariable logistic regression analysis showed that a history of diabetes (*P* < 0.001), prior antimicrobial use (*P* < 0.001), recurrent abscesses (*P* = 0.002), and length of hospital stay ≥14 days (*P* < 0.001) were statistically associated with MDR-positive cultures. However, the length of hospital stay was considered a postoperative process variable and should not be interpreted as a causal factor for MDR culture positivity. Patients in the MDR culture-positive group had longer incision healing times, prolonged antimicrobial use, higher hospitalization costs, and a higher incidence of complications compared with those in the non-MDR culture-positive group; all of these were unadjusted between-group associations.

**Conclusions:**

MDR culture positivity was relatively common among adult patients with perianal abscess undergoing postoperative wound culture and was associated with diabetes mellitus, prior antimicrobial use, and recurrent abscesses. Culture results should be interpreted in conjunction with clinical manifestations of infection. Treatment should be individualized based on pathogen-specific susceptibility results, patient conditions, and local antimicrobial resistance surveillance data. The observed associations should not be interpreted as causal inferences.

## 1 Introduction

Perianal abscess is a common acute infectious disease in colorectal surgery, and surgical incision and drainage are the main treatment methods (1–3). Perianal wounds are anatomically adjacent to the gastrointestinal tract and perineal region. Postoperative specimens may yield residual organisms from the primary abscess, wound contamination, or colonizers. Culture positivity alone does not necessarily indicate a clinically significant postoperative infection (4). Clinical infection should be diagnosed based on a comprehensive assessment of symptoms such as fever, worsening local redness, swelling, pain, or purulent drainage, elevated inflammatory markers, delayed wound healing, escalation of antimicrobial therapy, and repeat intervention. In recent years, multidrug-resistant organisms (MDRs) have posed challenges to the treatment of infections (5). However, in the postoperative setting of perianal abscess, it is particularly important to distinguish between MDR culture positivity and clinical MDR infection. Currently, available microbiological data regarding postoperative perianal abscess remain limited (6–8).

Therefore, this retrospective study aimed to describe the pathogenic characteristics of MDR strains isolated from postoperative wound culture in patients with perianal abscess and to explore patient and disease factors associated with MDR culture positivity. In this study, culture positivity alone was not considered equivalent to clinical infection, and causal inferences were not drawn from observational associations.

## 2 Materials and methods

### 2.1 Study design

This was a single-center, retrospective, observational case-control study. It was approved by the Ethics Committee of Xingtai People's Hospital, Affiliated Hospital of Hebei Medical University (approval number 2025233) and conducted in accordance with the requirements of the Declaration of Helsinki. Patients who underwent perianal abscess surgery in the Anorectal Surgery Department of our hospital between June 2022 and June 2025 were identified using the hospital information system and included in the study. All study participants were adult patients. Since this study involved the analysis of previously anonymized clinical data extracted from the hospital's electronic medical record system, the ethics committee waived the requirement for specific informed consent.

Sample size description: This was a retrospective study, and the sample size was primarily determined by the number of consecutive cases that met the inclusion and exclusion criteria within the study time window. Before the study, sample size estimation was performed based on previous studies on drug-resistant bacteria in perianal abscess and preliminary survey data from our hospital. The calculation assumed a two-sided *α* of 0.05, a power (1 − *β*) of 0.80, an MDR culture positivity rate of approximately 30%, an exposure-to-non-exposure ratio of about 1:2, and an expected OR of approximately 2.0 for the main risk factor. The estimated minimum sample size was 218 cases. We also followed the principle that about 10 outcome events per variable were required for multivariate logistic regression (9). This study ultimately included 246 patients, of whom 78 had MDR culture-positive results, which met the requirements for the primary analysis of this study.

The inclusion criteria were as follows: (1) patients aged ≥18 years; (2) patients diagnosed with perianal abscess who underwent surgical incision and drainage; (3) patients who underwent postoperative bacterial culture of wound exudate and antimicrobial susceptibility testing when clinically indicated; (4) patients with complete clinical data. It should be noted that the denominator in this study consisted of the 246 patients who met the above criteria and underwent postoperative cultures during the study period, rather than all patients who underwent perianal abscess surgery during the same period.

The exclusion criteria were as follows: (1) patients with severe immunodeficiency disorders; (2) patients with malignant tumors; (3) patients with preoperative systemic infections; (4) patients discharged or transferred within 48 h after surgery; (5) patients with missing bacterial culture results; (6) patients who underwent surgery at other sites.

After screening according to the above eligibility criteria, 246 patients were included in the study. Among them, 78 patients with MDR strains isolated from postoperative wound culture were assigned to the MDR culture-positive group, whereas 168 patients with non-MDR strains isolated from cultures were assigned to the non-MDR culture-positive group. Group allocation was based on microbiological findings and did not represent a clinical diagnosis of infection. Clinical infections and complications were diagnosed based on a comprehensive assessment of clinical findings, including local wound deterioration or purulent drainage, fever, elevated white blood cell count or C-reactive protein levels, escalation of antimicrobial therapy, repeat surgery or drainage, and delayed wound healing.

### 2.2 Surgical and specimen collection methods

All patients underwent spinal or sacral anesthesia and were placed in the lithotomy or lateral decubitus position. After routine disinfection and draping, incisions were made and adequate drainage was performed. When necessary, counter-drainage or seton drainage was performed. Dressings were changed daily postoperatively to maintain unobstructed drainage. The specimens used in this study were wound exudate samples collected 24–48 h postoperatively in accordance with the routine institutional protocol and sent for testing within 2 h. Since preoperative and intraoperative cultures were not systematically collected in our database, postoperative isolates could not be compared with the microbiota of the primary abscess on an individual basis. In addition, records of antibiotic exposure before specimen collection were incomplete in some cases. Therefore, the detected organisms may reflect residual organisms from the primary abscess, contamination, colonization, or true infection, and the findings should be interpreted in conjunction with clinical manifestations.

### 2.3 Bacterial culture and antimicrobial susceptibility testing

Specimens were inoculated onto blood agar and MacConkey agar plates and incubated at 35 °C for 24–48 h. Isolated strains were identified and subjected to antimicrobial susceptibility testing using an automated microbial identification and susceptibility analysis system. Susceptibility results were interpreted according to the Clinical and Laboratory Standards Institute (CLSI) criteria (10). MDR was defined as concurrent resistance to three or more different classes of antimicrobial agents. This definition was used to classify the antimicrobial resistance phenotype of isolated strains. The diagnosis of clinical infection was assessed based on a comprehensive evaluation of clinical manifestations.

### 2.4 Outcome measures

#### 2.4.1 Primary outcome measures

(1) Distribution of pathogens: the bacterial species identified in the MDR culture-positive group and their corresponding proportions were recorded; (2) Primary outcome: whether MDR strains were detected in postoperative wound culture; (3) Analysis of associated factors: univariable and multivariable logistic regression analyses were performed to identify factors associated with MDR culture positivity. Since the antimicrobial agents and interpretive breakpoints vary among bacterial species, overall resistance rates calculated from pooled strains were not used to guide clinical antimicrobial treatment decisions.

#### 2.4.2 Secondary outcome measures

(1) Baseline data: age, sex, body mass index (BMI), underlying diseases, smoking history, and alcohol use history were recorded; (2) Disease characteristics: abscess type, abscess location, abscess diameter, recurrence, and the presence of concomitant anal fistula were recorded; (3) Treatment-related factors: prior antimicrobial use, length of hospital stay, and operation time were recorded; (4) Prognostic indicators: incision healing time, duration of antimicrobial use, hospitalization costs, and occurrence of complications were recorded. Recurrence and postoperative complications were assessed during hospitalization and within 30 days after discharge, based on a combination of medical records, outpatient follow-up visits, and telephone follow-ups. Complications were assessed based on local wound presentation, body temperature, complete blood count, C-reactive protein, and other inflammatory markers, with imaging examinations or repeat bacterial cultures performed when necessary. Treatment failure, sepsis/severe persistent infection, and systemic inflammatory response syndrome were also included in the screening scope for complications.

### 2.5 Statistical methods

Data analysis was performed using SPSS 26.0 statistical software. Normally distributed continuous variables were expressed as mean ± standard deviation and analyzed using the independent samples *t*-test. Categorical data were expressed as number of cases and percentage [*n* (%)] and compared using the *χ*^2^-test or Fisher's exact test. MDR culture positivity in postoperative wound culture was used as the dependent variable. Variables with *P* < 0.05 in univariable analysis and clinically significant variables were included in the multivariable logistic regression model. Odds ratios (ORs) and 95% confidence intervals (CIs) were reported. Length of hospital stay ≥14 days was considered a postoperative process variable that may be influenced by MDR culture status. It was included in the original model solely for exploratory association analysis and should not be interpreted as a baseline predictor or a causal risk factor. Prognostic outcomes were only compared between unadjusted groups. A two-sided *P*-value of less than 0.05 was considered statistically significant.

## 3 Results

### 3.1 Comparison of baseline characteristics between the two groups

There were no statistically significant differences between the two groups in terms of sex, BMI, smoking history, and alcohol use history (*P* = 0.594, 0.410, 0.448, 0.462). However, the average age, the proportion of patients aged ≥60 years, the prevalence of diabetes, and the prevalence of hypertension in the MDR culture-positive group were all higher than those in the non-MDR culture-positive group (*P* = 0.001, 0.004, 0.001, 0.031) (Table 1).

**Table 1:** Comparison of baseline characteristics between the two groups (mean ± SD)/[*n* (%)].

Baseline characteristics	MDR culture-positive group (*n* = 78)	Non-MDR culture-positive group (*n* = 168)	t/*χ*^2^	P
Average age (years)	54.67 ± 12.35	48.23 ± 11.86	3.842	0.001
Age ≥60 years (cases)	34 (43.59)	42 (25.00)	8.526	0.004
Male/female (cases)	52/26	118/50	0.285	0.594
BMI (kg/m^2^)	24.56 ± 3.42	24.18 ± 3.28	0.826	0.410
Diabetes mellitus (cases)	32 (41.03)	35 (20.83)	10.854	0.001
Hypertension (cases)	28 (35.90)	38 (22.62)	4.628	0.031
Smoking history (cases)	26 (33.33)	48 (28.57)	0.576	0.448
Alcohol use history (cases)	22 (28.21)	40 (23.81)	0.542	0.462

BMI, body mass index; MDR, multidrug-resistant.

### 3.2 Distribution of pathogens in the MDR culture-positive group

A total of 96 pathogenic strains were isolated from 78 patients with MDR culture positivity, including 60 cases (76.92%) of single-pathogen infections and 18 cases (23.08%) of mixed infections. Among the isolated pathogens, 62 strains (64.58%) were Gram-negative bacteria and 34 strains (35.42%) were Gram-positive bacteria. *Escherichia coli* (*E. coli*) was the most commonly identified pathogen (36.46%), followed by *Staphylococcus aureus* (*S. aureus*) (22.92%), *Pseudomonas aeruginosa* (14.58%), and *Klebsiella pneumoniae* (11.46%) (Table 2). The above findings described the bacterial spectrum of postoperative wound culture and should not be interpreted as evidence of clinical infection alone.

**Table 2:** Distribution of pathogens in the MDR culture-positive group.

Pathogens		Strains	Composition ratio (%)
Gram-negative bacteria (*n* = 62, proportion of 64.58%)	*Escherichia coli*	35	36.46
	*Pseudomonas aeruginosa*	14	14.58
	*Klebsiella pneumoniae*	11	11.46
	*Proteus mirabilis*	2	2.08
Gram-positive bacteria (*n* = 34, proportion of 35.42%)	*Staphylococcus aureus*	22	22.92
	*Staphylococcus epidermidis*	6	6.25
	*Enterococcus faecalis*	4	4.17
	*Enterococcus faecium*	2	2.08

MDR, multidrug-resistant.

### 3.3 Comparison of disease characteristics between the two groups

The proportions of abscess diameter ≥5 cm, recurrent abscess, and concomitant anal fistula in the MDR culture-positive group were significantly higher than those in the non-MDR culture-positive group (*P* = 0.001, <0.001, 0.011). No statistically significant differences were observed between the two groups in terms of abscess type and abscess location (*P* > 0.05) (Table 3).

**Table 3:** Comparison of disease characteristics between the two groups.

Disease characteristics		MDR culture-positive group (*n* = 78) [n (%)]	Non-MDR culture-positive group (*n* = 168) [n (%)]	χ^2^	P
Abscess type	Perianal subcutaneous abscess	42 (53.85)	98 (58.33)	0.386	0.534
	Ischiorectal abscess	28 (35.90)	54 (32.14)		
	Pelvirectal abscess	8 (10.26)	16 (9.52)		
Abscess diameter ≥5 cm		46 (58.97)	62 (36.90)	10.524	0.001
Recurrent abscess		35 (44.87)	38 (22.62)	12.486	<0.001
Concomitant anal fistula		32 (41.03)	42 (25.00)	6.428	0.011

MDR, multidrug-resistant. Abscess diameter was categorized into two groups based on whether it was ≥5 cm; therefore, the chi-square test was used for comparison between groups.

### 3.4 Comparison of treatment-related factors between the two groups

The proportions of prior antimicrobial use and length of hospital stay ≥14 days in the MDR culture-positive group were significantly higher than those in the non-MDR culture-positive group (all *P* < 0.001). No statistically significant difference was observed in operation time between the two groups (*P* > 0.05) (Table 4).

**Table 4:** Comparison of treatment-related factors between the two groups (mean ± SD)/[*n* (%)].

Treatment-related factors	MDR culture-positive group (*n* = 78)	Non-MDR culture-positive group (*n* = 168)	t/χ^2^	P
Prior antimicrobial use (cases)	48 (61.54)	52 (30.95)	20.124	<0.001
Length of hospital stay (days)	16.82 ± 5.64	11.35 ± 4.28	8.124	<0.001
Length of hospital stay ≥14 days (cases)	52 (66.67)	48 (28.57)	31.256	<0.001
Operation time (min)	42.56 ± 12.84	40.23 ± 11.56	1.386	0.167
Intraoperative blood loss (mL)	35.42 ± 15.26	32.18 ± 13.84	1.624	0.106

MDR, multidrug-resistant.

### 3.5 Multivariable logistic regression analysis of factors associated with MDR culture positivity

**Figure 1:**
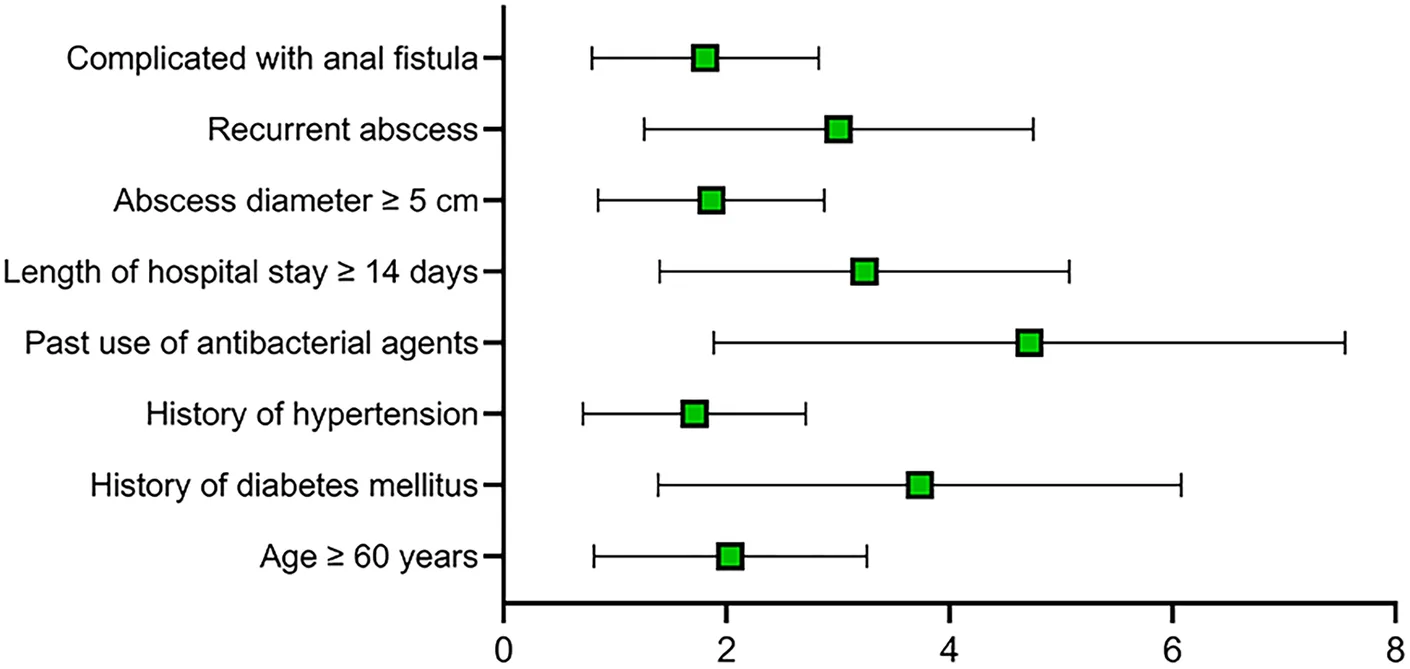
Multivariable logistic regression analysis of factors associated with MDR culture positivity. A history of diabetes (OR = 3.241, *P* < 0.001), prior antimicrobial use (OR = 4.150, *P* < 0.001), length of hospital stay ≥14 days (OR = 2.889, *P* < 0.001), and recurrent abscess (OR = 2.670, *P* = 0.002) were identified as independent risk factors for MDR culture positivity. MDR: multidrug-resistant; OR: odds ratio; 95% CI: 95% confidence interval. The horizontal axis represents the OR value, and the error line is the 95% CI.

Variables with statistical significance in the univariable analysis (age ≥60 years, a history of diabetes, a history of hypertension, abscess diameter ≥5 cm, recurrent abscess, concomitant anal fistula, prior antimicrobial use, and length of hospital stay ≥14 days) were included in the multivariable logistic regression analysis. The results showed that a history of diabetes (OR = 3.241, *P* < 0.001), prior antimicrobial use (OR = 4.150, *P* < 0.001), length of hospital stay ≥14 days (OR = 2.889, *P* < 0.001), and recurrent abscess (OR = 2.670, *P* = 0.002) were identified as independent risk factors for MDR culture positivity (Table 5, Figure 1). Among these factors, prior antimicrobial use had the highest OR, suggesting that it has the most significant impact on the risk of MDR culture positivity. Patients with a history of antimicrobial use had a risk of MDR culture positivity approximately 4.15 times higher than those without such history. The risk was about 3.24 times higher in patients with diabetes than in those without, about 2.89 times higher in those with length of hospital stay ≥14 days than in those with length of hospital stay <14 days, and about 2.67 times higher in patients with recurrent abscess than in those without recurrence.

**Table 5:** Multivariable logistic regression analysis of factors associated with MDR culture positivity.

Risk factor	B	S.E	OR (95% CI)	Wald	*P*
Age ≥60 years	0.582	0.318	1.789 (0.952–3.362)	3.286	0.070
History of diabetes	1.176	0.338	3.241 (1.672–6.285)	12.054	<0.001
History of hypertension	0.418	0.312	1.519 (0.826–2.794)	1.836	0.175
Prior antimicrobial use	1.423	0.321	4.150 (2.212–7.786)	19.685	<0.001
Length of hospital stay ≥14 days	1.061	0.302	2.889 (1.598–5.223)	12.486	<0.001
Abscess diameter ≥5 cm	0.518	0.289	1.679 (0.953–2.958)	3.212	0.073
Recurrent abscess	0.982	0.310	2.670 (1.456–4.895)	10.024	0.002
Concomitant anal fistula	0.482	0.298	1.619 (0.902–2.906)	2.568	0.109

MDR, multidrug-resistant; OR, odds ratio; SE, standard error; 95% CI: 95% confidence interval.

### 3.6 Comparison of prognostic indicators between the two groups

**Figure 2:**
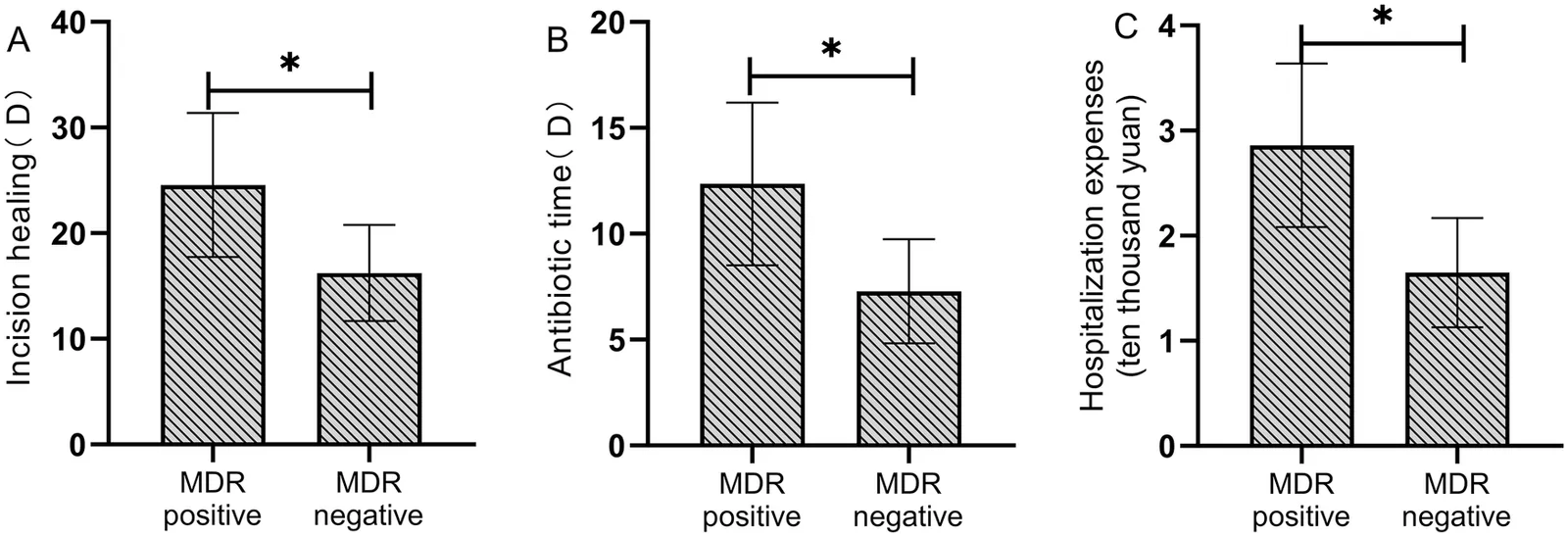
Comparison of prognostic indicators between the two groups. The incision healing time **(A)**, duration of antimicrobial use **(B)**, and hospitalization costs **(C)** of patients in the MDR culture-positive group were significantly higher than those in the non-MDR culture-positive group (*P* < 0.05). MDR: multidrug-resistant. *indicates statistically significant difference between groups. In **(A)**, the vertical axis represents incision healing time (days); in **(B)**, the vertical axis represents the duration of antimicrobial use (days); and in **(C)**, the vertical axis represents hospitalization costs (ten thousand Yuan).

The incision healing time [(24.18 ± 6.96) days vs. (16.03 ± 4.47) days], duration of antimicrobial use [(12.26 ± 3.78) days vs. (7.15 ± 2.54) days], and hospitalization costs [(2.82 ± 0.75) ten thousand yuan vs. (1.62 ± 0.51) ten thousand yuan] of patients in the MDR culture-positive group were significantly higher than those in the non-MDR culture-positive group (all *P* < 0.001) (Figure 2).

### 3.7 Comparison of postoperative complication rates between the two groups

**Figure 3:**
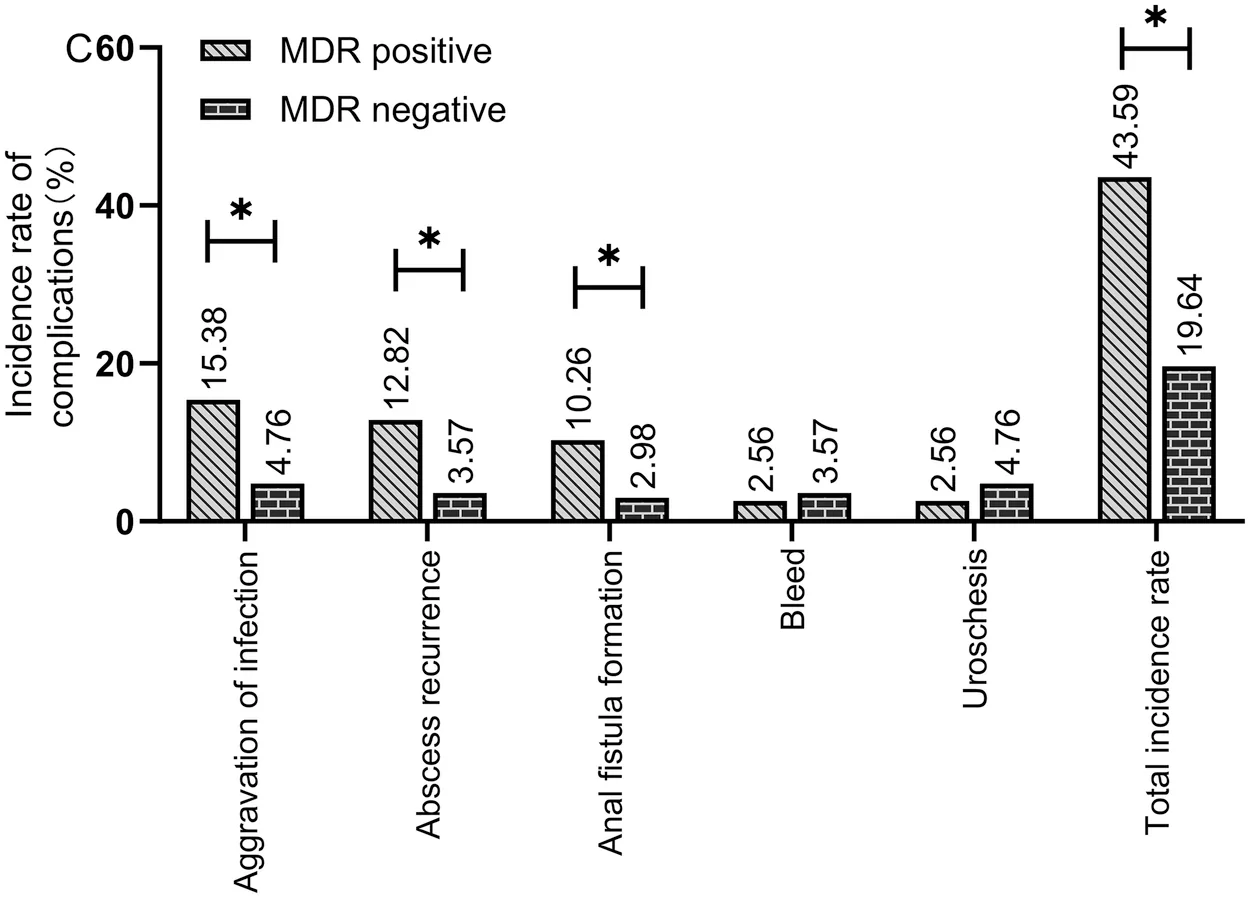
Comparison of postoperative complication rates between the two groups. The incidences of aggravated incision infection, abscess recurrence, and anal fistula formation were significantly higher in the MDR culture-positive group (*P* < 0.05). MDR: multidrug-resistant. *indicates statistically significant difference between groups. The values in the figure represent the incidence rate (%).

The overall incidence of postoperative complications in the MDR culture-positive group was 43.59% (34/78), which was higher than that in the non-MDR culture-positive group (19.64%, 33/168) (*P* < 0.001). Among them, the incidence of aggravated incision infection, abscess recurrence, and anal fistula formation was statistically significant (*P* < 0.05) (Figure 3). This comparison was based on unadjusted between-group differences and should not be interpreted as demonstrating that MDR culture positivity independently led to an increased risk of complications.

## 4 Discussion

### 4.1 Epidemiological characteristics of MDR culture positivity after perianal abscess surgery

In this study, MDR culture positivity was observed in 78 of 246 patients undergoing postoperative wound culture, with a detection rate of 31.71%. The denominator for this proportion consisted of patients who underwent postoperative wound culture and met the eligibility criteria, rather than all patients undergoing perianal abscess surgery during the same period. Therefore, this proportion should not be interpreted as the overall incidence of postoperative MDR infection. The perianal region is susceptible to influences from the primary abscess microbiota, fecal contamination, and colonization; therefore, culture results should be interpreted in conjunction with clinical symptoms, inflammatory markers, and the course of treatment (11, 12).

### 4.2 Pathogenic characteristics of MDR culture positivity

In this study, 96 strains of pathogens were isolated from 78 patients with MDR culture positivity, with a mixed infection rate of 23.08%. This is consistent with the clinical characteristics of perianal abscesses, including severe wound contamination and susceptibility to multiple bacterial infections. The pathogens isolated in this study were mainly Gram-negative bacteria, with *E. coli* accounting for 36.46%. This finding is consistent with the etiological characteristics of perianal abscesses, which are mainly caused by gut microbiota. Previous studies (13, 14) have indicated that *E. coli* is the dominant gut microbiota and the major ESBLs-producing species, and its antimicrobial resistance has become an increasingly concern. In this study, *Staphylococcus aureus* accounted for 22.92%, suggesting that in addition to focusing on enteric Gram-negative bacteria during the perioperative period, attention should also be paid to Gram-positive bacteria and potential sources of hospital-acquired infections (15). *Pseudomonas aeruginosa*, as an opportunistic pathogen, is more commonly isolated from patients with prolonged hospitalization and those receiving broad-spectrum antimicrobial treatment (16).

### 4.3 Analysis of factors associated with MDR culture positivity

Multivariable logistic regression analysis revealed that a history of diabetes, prior antimicrobial use, length of hospital stay ≥14 days, and recurrent abscess were identified as independent risk factors for MDR culture positivity after perianal abscess surgery, which was similar to the findings of previous studies (17–19). Specifically, due to long-term hyperglycemia, diabetic patients experience decreased immune function, microcirculatory disorders, and weakened tissue healing, thereby increasing susceptibility to infections that are difficult to control. This provides favorable conditions for the colonization and reproduction of drug-resistant bacteria, a point mentioned in previous studies (20, 21). Diabetes is an independent risk factor for various surgical site infections. In this study, prior antimicrobial use is the most important risk factor for MDR culture positivity. This may be related to selective pressure of antimicrobial agents. Frequent antimicrobial use may eliminate susceptible strains, but resistant bacteria gain a survival advantage and proliferate extensively (22–24). In this study, 61.54% of patients in the MDR culture-positive group had used antimicrobial agents within the past three months, significantly higher than 30.95% in the non-MDR culture-positive group. This result once again emphasizes the importance of the rational use of antimicrobial agents. Finally, although length of hospital stay ≥14 days was statistically significant in the original model, it may represent a consequence of MDR culture positivity, a surrogate marker of disease severity, or both, posing a significant risk of reverse causality. Therefore, this variable should not be interpreted as a baseline predictor (25–27). Patients with recurrent abscesses are relatively more prone to MDR culture positivity due to repeated surgeries, multiple uses of antimicrobial agents, and local tissue structure damage.

### 4.4 Impact of MDR culture positivity on prognosis

This study revealed that the incision healing time, the duration of antimicrobial use, and hospitalization costs of patients in the MDR culture-positive group were significantly higher than those in the non-MDR culture-positive group, and the incidence of postoperative complications was also significantly increased. Specifically, in the MDR culture-positive group, the incision healing time was (24.18 ± 6.96) days, the duration of antimicrobial use was (12.26 ± 3.78) days, and the hospitalization costs were (2.82 ± 0.75) ten thousand yuan, all of which were higher than those in the non-MDR culture-positive group. Due to the poor efficacy of conventional antibacterial drugs, MDR culture positivity often requires the use of higher-tier and expensive antimicrobial agents, and the treatment cycles tend to be prolonged. This not only increases the economic burden on patients but also affects their recovery process and quality of life. Furthermore, the increased incidence of various complications such as aggravated incision infection, abscess recurrence, and anal fistula formation further increases the disease burden on patients, and some patients may even require reoperation, ultimately affecting their prognosis (28).

### 4.5 Clinical implications and limitations

This study suggests that patients with diabetes, previous exposure to antibiotics, or recurrent abscesses should be subject to enhanced clinical monitoring. When clinical evidence of infection is present, specimens should be collected based on standard protocols, and treatment should be adjusted based on species-specific antimicrobial susceptibility results. Empirical antimicrobial therapy should be selected based on the severity of infection and local resistance surveillance. Unnecessary antimicrobial therapy should be de-escalated or discontinued as soon as possible. During the perioperative period, standardized drainage, dressing changes, and wound management should be emphasized. Recent research has also suggested that standardized wound irrigation and management may contribute to the prevention of surgical site infections (4).

This study also has some limitations. First, this was a single-center retrospective study that included only patients who underwent postoperative wound culture. Since the indications for culture were not pre-specified in the study, selection bias may have been introduced, and 31.71% represents only the MDR detection rate among the cultured cohort. Second, preoperative and intraoperative cultures were not systematically performed. Specimens collected 24–48 h postoperatively may reflect the microbiota of the primary abscess, residual contamination, or colonization. Therefore, culture positivity could not be considered equivalent to clinically significant infection. Third, records of antimicrobial exposure before specimen collection and information regarding the diagnosis of certain clinical infections were incomplete. Fourth, since susceptibility panels and interpretive breakpoints differ among bacterial species, the available aggregated data were insufficient to support reliable species-specific analyses of antimicrobial resistance. Fifth, postoperative variables such as length of hospital stay are susceptible to reverse causation, and comparisons of clinical outcomes were not adjusted for confounding factors. Finally, the relatively small sample size and limited long-term follow-up may restrict the generalizability of our findings. Future prospective studies should be conducted to standardize the culture indications and sampling times, compare paired preoperative/intraoperative and postoperative strains, and perform species-specific antimicrobial susceptibility testing and adjusted outcome analysis.

## 5 Conclusions

Among patients with perianal abscess who underwent postoperative wound culture at our institution, the rate of MDR culture positivity was 31.71%. *E. coli*, *S. aureus*, and *P. aeruginosa* were most frequently isolated pathogens. A history of diabetes mellitus, prior antimicrobial use, and recurrent abscess were associated with MDR culture positivity. Length of hospital stay was considered a postoperative process variable and should not be interpreted as a causal risk factor. MDR culture positivity was associated with longer wound healing time, increased use of antimicrobial agents, higher hospitalization costs, and a higher complication rate. However, causal relationships cannot be inferred from unadjusted comparisons. Culture results should be interpreted in conjunction with clinical evidence of infection, and treatment should be based on species-specific antimicrobial susceptibility results and local resistance surveillance.

## Data Availability

The original contributions presented in the study are included in the article/Supplementary Material, further inquiries can be directed to the corresponding author.
