# Correction: Pathogenic characteristics and factors associated with multidrug-resistant bacterial infections after perianal abscess surgery

**DOI:** 10.3389/fsurg.2026.1987070

**Published:** 2026-09-16

**Authors:** Bingjie Wang, Xujing Wei, Wanli Ma, Xiangsheng Wang, Binghui Hu, Yefang Zhao

**Affiliations:** 1 Department of Proctology, Xingtai People’s Hospital, Affiliated Hospital of Hebei Medical University, Xingtai, Hebei, China; 2 Department of Obstetrics and Gynecology, First Hospital of Hebei Medical University, Shijiazhuang, Hebei, China; 3 Department of Obstetrics and Gynecology, Xingtai People’s Hospital, Affiliated Hospital of Hebei Medical University, Xingtai, Hebei, China

**Keywords:** associated factor, multi-drug resistant bacteria, perianal abscess, retrospective study, wound culture

An incorrect Funding statement was provided. The correct funding statement reads:

This work was supported by the Xingtai Key Research and Development Program (Self-funded Project, Project No. 2025ZC351).

The original version of this article has been updated.

